# Helicobacter pylori

**DOI:** 10.3201/eid3208.251516

**Published:** 2026-08

**Authors:** Hellen C.O. Santos-Dutra, Caroline C.P. da Costa, Rodrigo S. Santos, Mônica S. Barbosa

**Affiliations:** Federal University of Goiás, Goiânia, Brazil

**Keywords:** Helicobacter pylori, bacteria, gastropathy, etymology, food safety

## Helicobacter pylori **[hel″i-kō-bak′tər pī′lor-ē]**

*Helicobacter pylori*, a gram-negative, spiral-shaped, microaerophilic bacterium, infects more than half the world’s population. The name derives from *helix* (spiral), *bacter* (bacterium), and *pylori* (Greek for gatekeeper, referring to the pylorus) (Figure). Discovered in 1982 by Barry Marshall and Robin Warren at Royal Perth Hospital in Australia, *H. pylori*’s role in gastritis was proven when Marshall ingested an inoculum. The scientist stated, “The only person in the world at that time who could make an informed consent about the risk of swallowing the *Helicobacter* was me.” In 2005, Warren and Marshall won the Nobel Prize in Physiology or Medicine for discovering that *H. pylori* causes gastropathies.

**Figure:**
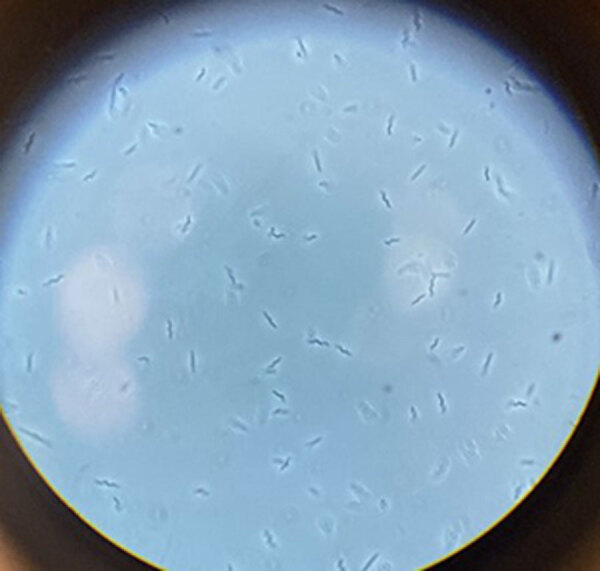
Representative electron micrograph of *Helicobacter pylori*, showing its spiral shape and polar flagella, which enable motility in the gastric mucus. Original magnification ×1,000. Photograph provided by the authors.
